# Nutritional assessment of Chinese gynecologic cancer survivors with post-surgical lower limb lymphedema: a cross-sectional study

**DOI:** 10.3389/fnut.2024.1484017

**Published:** 2024-10-28

**Authors:** Xiaoling Zhu, Xinjun Liu, Jinbo Tian, Chunshui He, Shan Huang

**Affiliations:** ^1^Department of Vascular Surgery, Chengdu DongLi Hospital, Chengdu, Sichuan, China; ^2^Department of Vascular Surgery, Hospital of Chengdu University of Traditional Chinese Medicine, Chengdu, Sichuan, China; ^3^Department of Oncology, Sichuan Provincial People's Hospital, Chengdu, Sichuan, China

**Keywords:** gynecologic cancers, lower limb lymphedema, nutritional status, serum albumin, Prognostic Nutritional Index

## Abstract

**Objective:**

This study aims to evaluate the nutritional status of Chinese gynecologic cancer survivors with post-surgical lower limb lymphedema (LLL) by analyzing serum albumin levels and the prognostic nutritional index (PNI).

**Methods:**

A retrospective cross-sectional study was conducted at two Chinese medical centers. The cohort comprised 155 gynecologic cancer survivors who developed symptomatic unilateral LLL post-surgery, during the period from September 2021 to June 2024. Nutritional status was assessed by measuring serum albumin and PNI. Statistical analysis was performed using univariate and multivariate logistic regression models to identify factors associated with low serum albumin and PNI.

**Results:**

The mean age of the cohort was 55.47 ± 10.56 years, and the median total survival time was 72.0 months (36.5, 111.5 months). The prevalence of low serum albumin (< 40 g/L) was 40.0%, and low PNI (< 45) was 80.6%. Significant associations were found between low hemoglobin levels and both low serum albumin (OR = 1.05, 95% CI: 1.02–1.08, *p* < 0.001) and low PNI (OR = 1.09, 95% CI: 1.04–1.13, *p* < 0.001). Advanced International Society of Lymphology stage 3 was also associated with low albumin (OR = 0.18, 95% CI: 0.03–0.99, *p* < 0.05).

**Conclusion:**

The study highlights a significant prevalence of high risk of malnutrition among Chinese gynecologic cancer survivors with LLL, underscoring the need for regular nutritional assessments and interventions.

## Introduction

Gynecological cancers, including cervical, endometrial, and ovarian cancers, represent a significant burden on women's health worldwide (1–3). Lower limb lymphedema (LLL), a common complication characterized by the accumulation of lymphatic fluid in the interstitial tissue of lower extremity, often occurs following radical surgery and radiotherapy (4–6). This condition leads to chronic swelling, pain, and decreased quality of life (7). The prevalence of LLL in gynecological cancer survivors ranges from 20% to 40%, according to previous studies. LLL remains a significant challenge, requiring long-term management and exacerbating the substantial difficulties faced by cancer survivors (6, 8, 9).

Nutritional status plays a pivotal role in the overall prognosis and quality of life for cancer patients. High risk of malnutrition is prevalent among cancer patients and is associated with increased morbidity, mortality, and healthcare costs (10, 11). Despite the well-recognized importance of nutritional status in cancer care, there is a notable lack of research focusing on the nutritional challenges faced by gynecological cancer survivors with LLL post-surgery. Current literature inadequately addresses the prevalence and impact of high risk of malnutrition in this specific patient population, underscoring the need for targeted studies to fill this deficit.

Serum albumin, a major plasma protein synthesized by the liver, serves as a marker for protein-energy malnutrition and systemic inflammation (12). Low serum albumin levels are indicative of poor nutritional status and have been linked to worse clinical outcomes (13). The Prognostic Nutritional Index (PNI), which combines serum albumin levels and lymphocyte count, provides a comprehensive assessment of both nutritional and immune status (14, 15). Both serum albumin and PNI has been widely used to evaluate the risk of postoperative complications, disease progression, and survival in various cancers (16, 17).

We aim to address the gap in the literature by evaluating the serum albumin and the PNI of patients who have undergone surgery for gynecological cancers and developed LLL. By retrospectively analyzing the patients from two Chinese medical centers, we seek to identify significant associations and provide insights into the presence of high risk of malnutrition in this vulnerable patient population. We hypothesize that high risk of malnutrition, indicated by low serum albumin and PNI levels, is prevalent among gynecologic cancer survivors with LLL, and addressing these nutritional challenges could improve their overall prognosis and quality of life.

## Materials and methods

### Study design

We conducted a retrospective cross-sectional study to assess the nutritional status of patients with gynecological cancer who developed symptomatic unilateral LLL after surgery and were subsequently referred for intensive complex decongestive lymphatic therapy (CDT) at two Chinese hospitals.

### Patient selection and data collection

The data from all patients admitted to hospitals who underwent CDT between September 2021 and June 2024 were collected using a dedicated LLL clinical research system and subsequently analyzed. This study adhered to the principles of the Declaration of Helsinki. The local ethics committee granted approval for the study (protocol number: 2021KL-076), and all patients provided their written informed consent upon their admission into the study.

The diagnosis of LLL was made in accordance with the 2020 consensus document of the International Society of Lymphology (ISL) (18). It was based on a comprehensive evaluation of the patient's medical history, physical examination, and ultrasound scan of the lower limb subcutaneous tissue upon admission. The diagnostic criterion for LLL included an interlimb volume difference exceeding 10% when compared to the contralateral limb. Additionally, the presence of a subcutaneous echo-free space observed in ultrasound scans was indicative of LLL.

The study's inclusion criteria included: women who had received surgical treatment for gynecological cancer; developed unilateral LLL at least 12 months after surgery (18). Exclusion criteria included: cancer recurrence or metastasis; deep venous thrombosis or chronic venous insufficiency; infection on the edematous limb; presence of other malignancies; chronic liver and kidney dysfunction.

### LLL measures

The severity of lymphedema was classified based on the ISL staging system (mild: stages 0 to I; moderate-to-severe: stages II to III) (18). The circumference of both lower extremities was measured at a total of five locations (knee, 10 and 30 cm above and below the knee). The formula used to calculate the volume of a lower extremity segment was derived by Casley-Smith (19). It is represented as V=h(C^2^+Cc+c^2^)/12π, where V denotes the volume, C and c represent the circumferences at each end, and h indicates the distance between the ends. The severity of lymphedema is defined as the percentage of excess volume (PEV), the excess volume [the difference between lymphedema leg (VL) and healthy leg (VH) relative to the healthy leg volume (VH)], PEV = (baseline VL – VH)/VH × 100%. The PEV is more effective in defining the severity of lymphedema compared to the absolute difference in volume (20). Additionally, in this study, we focus on unilateral LLL and exclude bilateral LLL for this reason. The PEV of the entire leg, lower leg, and upper leg were all calculated.

### Nutrition status assessment and definition

The nutritional status of patients was evaluated by measuring serum albumin and the PNI. Prior to treatment, peripheral venous blood was collected from all patients, and serum albumin, total protein, and complete blood cell counts were assessed using colorimetry with an automatic biochemical analyzer (Beckman, USA). In this study, we adopted the diagnostic criteria as follows: Serum albumin: Normal range: 40–55 g/L; Low albumin: Albumin < 40 g/L. PNI: PNI = Serum albumin (g/L) + 0.005 × lymphocyte count per microliter (14). Low PNI was defined as the PNI < 45.

### Statistical analysis

Continuous variables were described using the mean ± standard deviation (SD) or media (interquartile 25, interquartile 75), depending on their distribution as assessed by the Shapiro-Wilk test. Categorical variables were presented as frequencies (*n*) and percentages (%). For the univariate analysis, the independent t-test was used to compare continuous variables when data were normally distributed, while the Mann-Whitney U test was applied for non-normally distributed data. Categorical variables were analyzed using the Chi-square test or Fisher's exact test as appropriate to evaluate associations between groups. Venn diagrams were used to illustrate the relationship between serum albumin and PNI.

Multivariate analysis was conducted using logistic regression models to identify independent predictors of low serum albumin and low PNI. Variables with a p-value < 0.1 in the univariate analysis were entered into the multivariate model to control for confounding factors. Adjusted odds ratios (OR) and 95% confidence intervals (CI) were calculated to quantify the strength of associations. A p-value of < 0.05 was considered statistically significant. All statistical tests were two-sided, and analyses were performed using R version 4.0.2 (R Foundation for Statistical Computing, Vienna, Austria).

## Results

### Patient characteristics

**Figure 1:**
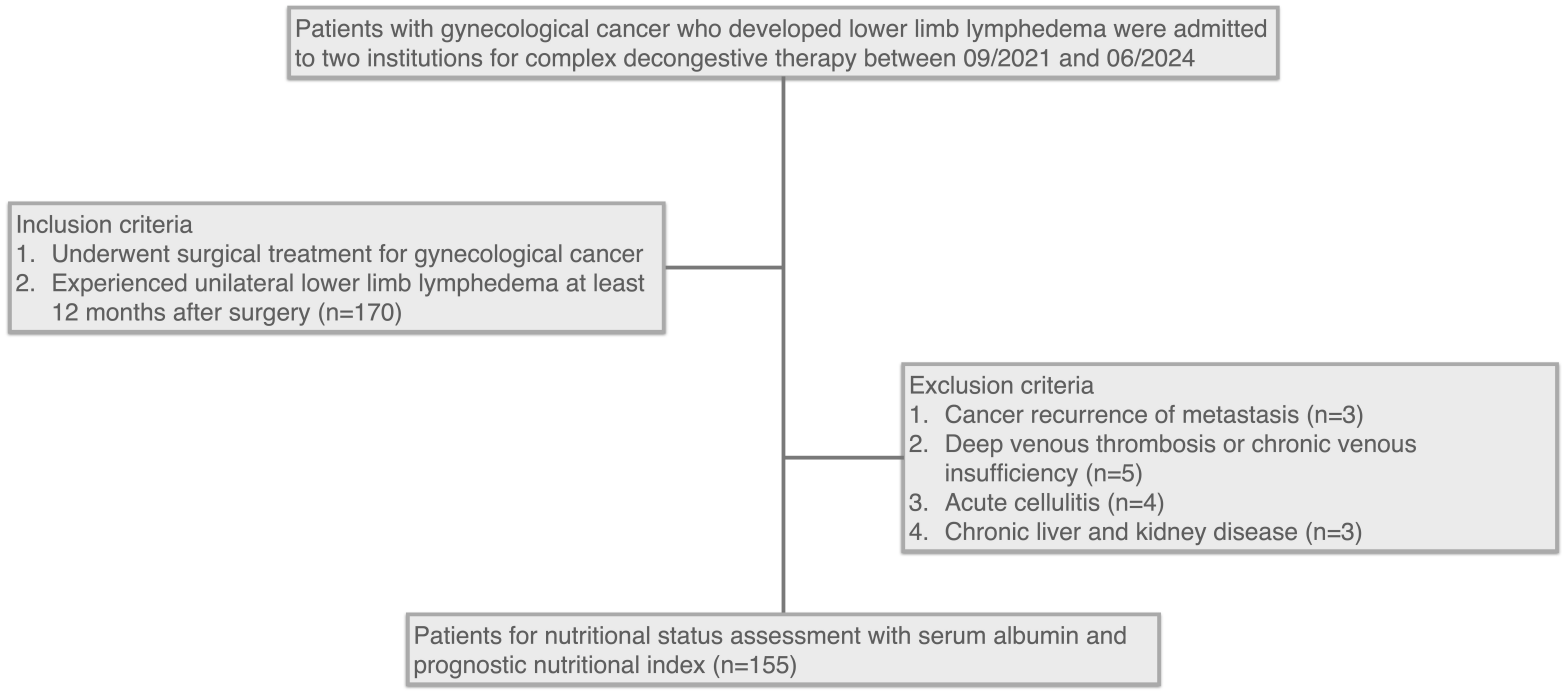
Flowchart of patient selection for nutritional status assessment in gynecological cancer survivors with lower limb lymphedema.

A total of 155 patients were included in this study (Figure 1). The mean age of the cohort was 55.47 ± 10.56 years, and the mean Body Mass Index (BMI) was 26.26 ± 4.06. Laboratory tests showed a median hemoglobin level of 135 g/L (125, 145 g/L), a mean white cell count of 4.89 ± 1.35 x 10^9^/L, and a median neutrophil percentage of 61.00% (56.45, 66.05%). The cohort included 104 (67.10%) patients with cervical cancer, 41 (26.45%) with endometrial cancer, and 10 (6.45%) with ovarian cancer.

Post-surgical treatment modalities included chemotherapy in 111 (72.61%) patients and radiotherapy in 108 (69.68%) patients. The median time to onset of lower limb lymphedema (LLL) following surgery was 36.0 months (12.0, 64.5 months), with a median edema duration of 24.0 months (12.0, 57.5 months), and a median total survival time of 72.0 months (36.5, 111.5 months). Sixteen patients (10.32%) had a history of cellulitis. LLL was left-sided in 81 (52.26%) patients and right-sided in 74 (47.74%) patients. According to ISL staging, 9 (5.81%) patients were at stage 1, 109 (70.32%) at stage 2, and 37 (23.87%) at stage 3. Edema quantification using PEV revealed median values of 41.58% (24.90, 63.47%) for the lower leg, 34.20% (22.03, 51.79%) for the upper leg, and 38.15% (24.38, 51.60%) for the whole leg. Detailed characteristics are summarized in Table 1.

**Table 1:** Patient characteristic and nutritional status.

**Characteristics**	**Value (*n* = 155)**	**Serum albumin**			**Prognostic nutrition index (PNI)**		
		**Low**,<**40;** ***N*** = **62 (40.0%)**	**Normal**, ≥**40;** ***N*** = **93 (60.0%)**	***P*** **value**	**Low**,<**45;** ***N*** = **125 (80.6%)**	**Normal**, ≥**45;** ***N*** = **30 (19.4%)**	***p*** **value**
Age	55.47 ± 10.56	57.21 ± 11.55	54.31 ± 9.74	0.11	55.79 ± 10.56	54.13 ± 10.65	0.44
BMI	26.26 ± 4.06	26.10 (23.47,29.25)	25.08 (23.44,28.89)	0.52	25.89 (23.46,29.27)	25.84 ± 3.56	0.50
Hemoglobin	135 (125,145)	126.5 (120.25,135.75)	134.0 (126.0,141.0)	0.0012^*^	132 (125,140)	140 (133,150)	< 0.05^*^
White cell count	4.89 ± 1.35	4.85 ± 1.25	4.92 ± 1.42	0.76	4.94 ± 1.36	4.71 ± 1.31	0.41
Neutrophil percentage (%)	61.00 (56.45,66.05)	61.12 (58.10,64.60)	60.90 (55.40,66.58)	0.78	61.00 (57.30, 65.70)	61.12 (53.67, 67.70)	0.77
**Type of cancer**							
Cervical	104 (67.10%)	47 (75.81%)	57 (61.29%)	0.06	87 (69.60%)	17 (56.67%)	0.17
Endometrial	41 (26.45%)	14 (22.58%)	27 (29.03%)		32 (25.60%)	9 (30.00%)	
Ovarian	10 (6.45%)	1 (1.61%)	9 (9.68%)		6 (4.80%)	4 (13.33%)	
Post-surgery time	36.0 (12.0, 64.5)	36.0 (12.0,81.0)	33.0 (12.0,60.0)	0.60	36.00 (12.00, 66.00)	24.00 (12.00, 60.00)	0.50
Edema time	24.0 (12.0, 57.5)	36.0 (12.0,48.0)	24.0 (12.0,60.0)	0.59	25.00 (12.00, 60.00)	22.00 (12.00, 48.00)	0.80
Survival time	72.0 (36.5, 111.5)	78.0 (38.75,124.0)	72.0 (36.0,101.0)	0.45	72.00 (40.00, 110.00)	66.00 (30.00, 117.00)	0.47
Chemotherapy	111 (71.61%)	46 (74.19%)	65 (69.89%)	0.69	89 (71.20%)	22 (73.33%)	0.99
Radiotherapy	108 (69.68%)	46 (74.19%)	62 (66.67%)	0.41	86 (68.80%)	22 (73.33%)	0.79
Infection history	16 (10.32%)	7 (11.29%)	9 (9.68%)	0.79	13 (10.40%)	3 (10.00%)	1.00
**Side of lower limb**							
Left edema	81 (52.26%)	30 (48.39%)	51 (54.84%)	0.53	65 (52.00%)	16 (53.33%)	1.00
Right edema	74 (47.74%)	32 (51.61%)	42 (45.16%)		60 (48.00%)	14 (46.67%)	
**ISL stage**							
1	9 (5.81%)	2 (3.23%)	7 (7.53%)	0.042^*^	5 (4.00%)	4 (13.33%)	0.14
2	109 (70.32%)	39 (62.90%)	70 (75.27%)		89 (71.20%)	20 (66.67%)	
3	37 (23.87%)	21 (33.87%)	16 (17.20%)		31 (24.80%)	6 (20.00%)	
Lower leg PEV (%)	41.58 (24.90,63.47)	46.82 (29.89,67.41)	38.19 (20.87,57.56)	0.047^*^	43.38 (25.93, 64.58)	32.82 (19.74, 52.29)	0.20
Upper leg PEV (%)	34.20 (22.03,51.79)	38.52 (22.41,54.35)	30.55 (21.57,47.24)	0.22	36.27 (22.48, 52.32)	29.63 (14.43, 38.67)	0.09
Whole leg PEV (%)	38.15 (24.38,51.60)	41.96 (27.52,60.89)	35.04 (22.67,47.17)	0.07	38.50 (25.89, 54.65)	32.00 (19.49, 41.96)	0.06

Continuous variables were described using the mean ± standard deviation (SD) or media (interquartile 25, interquartile 75), depending on their distribution as assessed by the Shapiro-Wilk test. Categorical variables were presented as frequencies (n) and proportions (%).

^*^P < 0.05.

ISL, International Society of Lymphology.

PEV, percentage of excess volume.

### Nutritional status of the patients

Table 1 also details the prevalence of low albumin and PNI among patients and compares clinical features between low and normal albumin and PNI groups.

Analysis using albumin < 40 g/L as a cutoff indicated that 62 (40.0%) patients had low serum albumin levels. When using PNI < 45 as a cutoff, 125 (80.6%) patients had low PNI scores. Comparison between the low albumin group and the normal group revealed statistically significant differences in hemoglobin levels, ISL stage, and lower leg PEV (*p* < 0.05). However, only hemoglobin levels (p < 0.05) showed a significant difference between low PNI and normal PNI groups.

**Figure 2:**
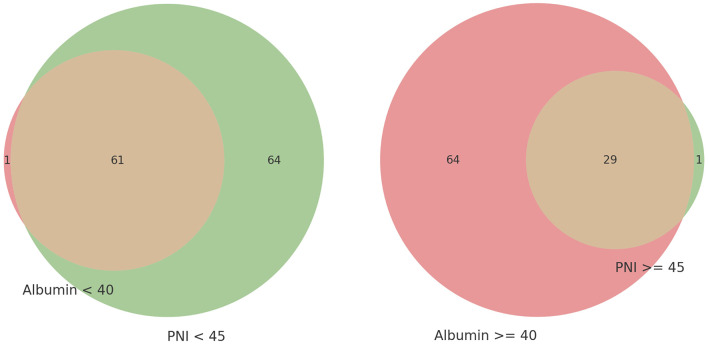
Venn diagram of nutritional status evaluation by low serum albumin level and prognostic nutritional index (PNI).

Figure 2 presents a Venn diagram comparing two methods of evaluating the same cohort. It highlights that patients with albumin levels below 40 g/L were mostly included in the PNI < 45 group, with only one exception. Similarly, patients with PNI ≥45 were predominantly in the albumin ≥40 g/L group, with only one exception. This demonstrates a strong concordance between albumin levels and PNI scores in assessing nutritional status, revealing significant overlap and notable differences within this specific patient population.

### Factor associated with low albumin and PNI

Variables associated with low albumin were identified using univariate logistic regression analysis. Variables with *p* < 0.1 between the low albumin group and the normal group included hemoglobin, type of cancer, ISL stage, lower leg PEV, and whole leg PEV. Hemoglobin and ISL stage were further tested using multivariate logistic regression. The results, shown in Table 2, demonstrated that low hemoglobin (OR = 1.05, 95% CI: 1.02-1.08, p < 0.001) and ISL stage 3 (OR = 0.18, 95% CI: 0.03-0.99, *p* < 0.05) were associated with low albumin.

**Table 2:** Factors associated with low serum albumin.

**Variables**	**Univariate**			**Multivariate**		
	β	***P***	**OR (95%CI)**	β	***P***	**OR (95%CI)**
Hemoglobin	0.04	0.002^*^	1.04 (1.02–1.07)	0.05	< 0.001^*^	1.05 (1.02–1.08)
**Cervical**						
0			1.00 (Reference)			
1	−0.68	0.062	0.51 (0.25–1.03)			
**Endometrial**						
0			1.00 (Reference)			
1	0.34	0.373	1.40 (0.67–2.95)			
**Ovarian**						
0			1.00 (Reference)			
1	1.17	0.144	3.21 (0.67–15.41)			
**ISL stage**						
1			1.00 (Reference)			1.00 (Reference)
2	−0.67	0.419	0.51 (0.10–2.59)	−0.65	0.438	0.52 (0.10–2.70)
3	−1.52	0.079	0.22 (0.04–1.19)	−1.74	0.049^*^	0.18 (0.03–0.99)
Lower Leg PEV	−0.01	0.142	0.99 (0.98–1.00)			
Whole Leg PEV	−0.01	0.246	0.99 (0.98–1.01)			

OR, odds ratio; CI, confidence interval.

^*^P < 0.05.

ISL, International Society of Lymphology.

PEV, percentage of excess volume.

Variables associated with low PNI were identified using univariate and multivariate logistic regression analysis. Variables with *p* < 0.1 between the low PNI group and the normal group included hemoglobin, upper leg PEV, and whole leg PEV. The results, shown in Table 3, indicated that only low hemoglobin (OR = 1.09, 95% CI: 1.04-1.13, *p* < 0.001) was associated with low PNI.

**Table 3:** Factors associated with prognostic nutritional index (PNI).

**Variables**	**Univariate**			**Multivariate**		
	β	***P***	**OR (95%CI)**	β	***P***	**OR (95%CI)**
Hemoglobin	0.08	< 0.001^*^	1.09 (1.04–1.13)	0.08	< 0.001^*^	1.09 (1.04–1.13)
Upper Leg PEV	−0.01	0.195	0.99 (0.97–1.01)			
Whole Leg PEV	−0.01	0.209	0.99 (0.97–1.01)			

OR, odds ratio; CI, confidence interval.

^*^P < 0.05.

PEV, percentage of excess volume.

## Discussion

This study evaluated the nutritional status of Chinese gynecologic cancer survivors experiencing LLL by analyzing serum albumin levels and PNI. Our findings indicate a high prevalence of high risk of malnutrition among gynecologic cancer survivors with LLL, with 40.0% of patients having serum albumin levels below 40 g/L and 80.6% having PNI scores below 45. Notably, our study is the first to specifically document the nutritional deficiencies and their prevalence among gynecologic cancer survivors with LLL, providing new insights into an under-researched patient population and highlighting significant nutritional challenges faced by this patient group.

Our study corroborates previous research indicating that high risk of malnutrition is common among cancer patients. Numerous studies (21–23) have highlighted that low albumin levels are prevalent among gynecologic cancer patients and are often linked to poorer prognosis, increased postoperative complications, and reduced overall survival rates. Low serum albumin levels reflect both poor nutritional status and an ongoing inflammatory response, which are critical in the management and recovery of cancer patients (24, 25). The clinical implications include heightened susceptibility to infections, delayed wound healing, and prolonged hospital stays. Wang et al. in a recent meta-analysis confirmed the association between low albumin levels and adverse clinical outcomes in gynecologic cancer patients (26).

Research has identified the PNI as an important marker in assessing the nutritional status and prognosis of gynecologic cancer patients (27–29). The PNI, which combines serum albumin levels and total lymphocyte count, provides insight into a patient's nutritional and immune status, as well as their inflammatory state (30, 31). Low PNI scores are associated with worse clinical outcomes, including lower overall survival rates and increased risk of complications during and after treatment (32–34). Clinically, low PNI scores indicate a need for intensive nutritional support and close monitoring to improve patient outcomes (35). Wang, et al. in a comprehensive review emphasized the role of PNI as a significant prognostic factor and its utility in guiding nutritional interventions (36).

While many studies use albumin < 35 g/L as a cutoff, our study utilized < 40 g/L as the threshold, as our laboratory tests indicated this level as abnormal. According to a meta-analysis by Ge et al. 42% of studies used albumin < 35 g/L as the cutoff, while only 17% used < 40 g/L (37). Additionally, most studies using < 35 g/L involve patients either pre- or post-surgery (38, 39), whereas our cohort comprises long-term survivors with a median total survival time of 72.0 months (36.5, 111.5 months). Given the extended survival time of our patients, using a less stringent cutoff of < 40 g/L is more reasonable to accurately reflect their nutritional status. Conversely, PNI < 45 is considered a relatively higher threshold for diagnosing high risk of malnutrition. A meta-analysis revealed that the predominant PNI cutoff was above 45, employed in 50% of research, whereas a cutoff of 45 was utilized in just 22%, suggesting that < 45 represents a more stringent criterion (36). It was surprising that the low threshold for albumin combined with the higher threshold for PNI resulted in a much larger proportion of patients being classified as high risk of malnourish according to PNI criteria. This discrepancy may be due to the inclusion of lymphocyte count in PNI calculation, as lymphedema is a chronic inflammatory condition that can affect lymphocyte levels (40).

Univariate and multivariate regression analyses demonstrated that low hemoglobin levels (OR = 1.05, 95% CI: 1.02–1.08, *P* < 0.01) and ISL stage 3 (OR = 0.18, 95% CI: 0.03–0.99, *P* < 0.05) were associated with low albumin, while only low hemoglobin (OR = 1.09, 95% CI: 1.04–1.13, *P* < 0.001) was associated with low PNI. The association between low hemoglobin and low albumin is expected, as hemoglobin is another nutrition index closely related to albumin (41). Low hemoglobin levels indicate anemia, which often coincides with poor nutritional status. This relationship is well-documented in gynecologic cancer research, where anemia frequently accompanies malnutrition and inflammation (42). This finding is consistent with research by Wang et al. which highlights the correlation between hemoglobin and nutritional deficiencies, reinforcing this link (26). The link between ISL stage 3 and low albumin suggests that advanced lymphedema, as a chronic inflammatory condition, exacerbates nutritional deficiencies. This association analysis is also consistent with previous findings by Mukai et al. which demonstrated that chronic inflammation negatively impacts nutritional status and leads to lower albumin levels (43).

Although it is well known that low albumin levels and PNI scores serve as prognostic predictors for overall survival rates, studies focusing on cancer survivors with LLL are rare. Considering that the cohort of this study consists of long-term cancer survivors, it is important to note that lymphedema itself can influence albumin levels and PNI scores. The chronic inflammation associated with LLL may exacerbate nutritional deficiencies, making it unclear how albumin levels and PNI scores will impact overall survival rates in this specific cohort. Therefore, while low albumin and PNI are established markers for poor prognosis in general cancer populations, their prognostic value in long-term cancer survivors with LLL remains uncertain and warrants further investigation. Future studies should aim to elucidate the specific impact of these nutritional markers on the survival outcomes of this unique patient population.

Our findings have important clinical implications. Given the high prevalence of low albumin and PNI among gynecologic cancer survivors with LLL, regular nutritional assessments should be integrated into their care plans. Interventions aimed at improving nutritional status, such as dietary modifications and nutritional supplementation, could help mitigate the adverse effects of malnutrition and improve overall prognosis and quality of life.

## Study limitations

While this study provides valuable insights into the nutritional status of gynecologic cancer survivors with LLL, several limitations must be acknowledged. First, the retrospective design introduces potential selection bias, as patient data were collected from only two medical centers, which may limit the generalizability of the findings. The study population was limited to Chinese gynecologic cancer survivors, and the results may not be fully applicable to other populations with different ethnic, geographic, or clinical characteristics.

Additionally, the reliance on serum albumin and PNI as primary markers of nutritional status may not capture the full spectrum of nutritional deficiencies, particularly in patients with complex cancer-related conditions. Serum albumin can be influenced by factors unrelated to nutrition, such as inflammation or liver function, which could confound the associations observed in this study.

Future studies should aim to validate these findings across larger, more diverse populations to enhance the generalizability of the results. Moreover, research should focus on assessing the efficacy of targeted nutritional interventions, such as dietary modifications and nutritional supplementation, in improving clinical outcomes for gynecologic cancer survivors with LLL. Longitudinal studies are also needed to clarify the long-term effects of nutritional status on patient prognosis, quality of life, and survival rates in this vulnerable population.

## Conclusions

This study highlights the significant prevalence of high risk of malnutrition among Chinese gynecologic cancer survivors with LLL, underscoring the need for regular nutritional assessments and interventions. Addressing the nutritional challenges faced by this vulnerable patient population can help improve their overall prognosis and quality of life.

## Data Availability

The raw data supporting the conclusions of this article will be made available by the authors, without undue reservation.
